# Molecular mechanism study of HGF/c-MET pathway activation and immune regulation for a tumor diagnosis model

**DOI:** 10.1186/s12935-021-02051-2

**Published:** 2021-07-14

**Authors:** Zhibo Shen, Wenhua Xue, Yuanyuan Zheng, Qishun Geng, Le Wang, Zhirui Fan, Wenbin Wang, Ying Yue, Yunkai Zhai, Lifeng Li, Jie Zhao

**Affiliations:** 1 Department of Pharmacy, The First Affiliated Hospital of Zhengzhou University, Zhengzhou, 450052 Henan People’s Republic of China; 2 Cancer Center, The First Affiliated Hospital of Zhengzhou University, Zhengzhou, 450052 Henan People’s Republic of China; 3 Department of Otorhinolaryngology, The First Affiliated Hospital of Zhengzhou University, Zhengzhou, 450052 Henan People’s Republic of China; 4 Department of Traditional Chinese Medicine, The First Affiliated Hospital of Zhengzhou University, Zhengzhou, 450052 Henan People’s Republic of China; 5 Internet Medical and System Applications of National Engineering Laboratory, Zhengzhou, China; 6 Department of clinical laboratory, The No.7.People’s Hospital of Zhengzhou, Zhengzhou, 450016 Henan China

**Keywords:** HGF, c-MET, RNAseq, Immune cells, Pathway score, Immune infiltration, Diagnostic predictor, Prognosis

## Abstract

**Background:**

Hepatocyte growth factor (HGF) binds to the c-mesenchymal-epithelial transition (C-MET) receptor and activates downstream signaling pathways, playing an essential role in the development of various cancers. Given the role of this signaling pathway, the primary therapeutic direction focuses on identifying and designing HGF inhibitors, antagonists and other molecules to block the binding of HGF to C-MET, thereby limiting the abnormal state of other downstream genes.

**Methods:**

This study focuses on the analysis of immune-related genes and corresponding immune functions that are significantly associated with the HGF/c-MET pathway using transcriptome data from 11 solid tumors.

**Results:**

We systematically analyzed 11 different cancers, including expression correlation, immune infiltration, tumor diagnosis and survival prognosis from HGF/c-MET pathway and immune regulation, two biological mechanisms having received extensive attention in cancer analysis.

**Conclusion:**

We found that the HGF/c-MET pathway affected the tumor microenvironment mainly by interfering with expression levels of other genes. Immune infiltration is another critical factor involved in changes to the tumor microenvironment. The downstream immune-related genes activated by the HGF/c-MET pathway regulate immune-related pathways, which in turn affect the degree of infiltration of immune cells. Immune infiltration is significantly associated with cancer development and prognosis.

**Supplementary Information:**

The online version contains supplementary material available at 10.1186/s12935-021-02051-2.

## Background

The c-mesenchymal-epithelial transition (c-MET) is a kinase receptor for hepatocyte growth factor (HGF), and has been proved to be a crucial factor in driving tumorigenesis [1–3]. The binding of HGF and c-MET triggers several downstream signaling pathways such as phosphoinositide 3-kinase/threonine-protein kinase (PI3K/AKT) pathway, wingless-related integration site (Wnt) pathway, and other tumor-related functions [4–6]. Eventually, the tumor microenvironment (TME) is transformed into a more suitable condition for tumor aggressiveness.

The HGF/c-MET receptor tyrosine kinase (RTK) pathway is inactive in normal tissues but active in various tumors [7]. An increasing number of studies have confirmed that inhibition of HGF/c-MET signaling is an effective therapeutic strategy for suppression of multiple human cancers, such as non-small cell lung cancer (NSCLC), hepatocellular carcinoma (HCC), gastric cancer, colorectal cancer, ovarian cancer, bladder cancer, head and neck cancer and cervical cancer [2, 8–14]. In preclinical and clinical trials, it has been demonstrated that c-MET inhibitors exhibit antitumor activity in the treatment of multiple types of cancers, especially in NSCLC. Moreover, in epidermal growth factor receptor tyrosine kinase inhibitor (EGFR-TKI)-resistant and EGFR-TKI-naive NSCLC patients, a combination of c-MET inhibitors and EGFR-TKIs (EGFR inhibitors) may be considered as a promising treatment option [15]. Based on its critical role in tumor progression, c-Met is emerging as a therapeutic target for cancer therapy. Treatment strategies in clinical trials include small molecule inhibitors specific to the tyrosine kinase domain of c-Met and monoclonal antibodies against HGF [16].

Tumor tissues are often infiltrated by a variety of immune cells such as T and B lymphocytes, natural killer (NK) cells, NK-T cells, dendritic cells (DCs), macrophages, neutrophils, eosinophils and mast cells. The TME contains numerous immune and inflammatory cells originating from lymphoid precursors, of which each type has a preferred location within the tumor site. Cytotoxic T-lymphocytes (CTLs) and Th1 cells are generally located at the boundary or core of tumor tissues. Naive DCs are commonly found in the core site of tumor tissues, whereas mature DCs infiltrate T-cell zones enriched with CD4+ and CD8+ T-cells. B-cells are more commonly distributed in tertiary lymphoid structures (TLS). Tumor-associated macrophages (TAMs) and T follicular helper cells (TFH) are found within B-cell zones, while NK cells are scattered within the stroma and at the tumor margins [17]. Based on the specific distribution, it can be speculated that the infiltration of different immune cells varies across different types of tumors. Besides, even in the same kind of cancer, the infiltration level of immune cells also changes due to the tumor heterogeneity. As tumor cells proliferate and metastasize, the immune cells also exhibit different behaviors. Numerous studies have confirmed that immune cell infiltration is significantly associated with cancer prognosis. Recent research highlights the prominent function of memory T cells [18] and CD8 T cells [19] in predicting patients’ prognosis regarding survival time. Therefore, the immune infiltration in different tumors is a critical factor in assessing tumor progression and predicting tumor prognosis.

To systematically study the complex regulation of the HGF/c-MET pathway and immune infiltration during the occurrence and development of tumors, we integrated the HGF/c-MET activation pathway and immune regulation-related pathways. By investigating the expression profiles of HGF and c-MET in all tumors in the TCGA database, we selected 11 solid tumors with significant differences in HGF or c-MET expression between tumor and normal tissues. Our first challenge was to distinguish between HGF/c-MET-activated and -inactivated samples within the 11 different cancers. We were unable to verify the experimental level for each sample, but the expression levels of the two genes were considered relevant in HGF/c-MET-activated samples. Therefore, we selected HGF/c-MET expression-correlated samples as the activated group samples and the rest as the inactive group samples. Next, we extracted the immune-related genes differentially expressed between activated and inactivated HGF/c-MET pathway through differential analysis. By evaluating the immune scores of immune-related functions and the infiltration scores of immune cells, we compared the differences before and after HGF/c-MET activation at immune levels. Finally, we constructed a diagnostic model featuring immune cells and immune-related pathways. We found it difficult to distinguish between tumor samples and normal samples when using HGF, c-MET, or immune infiltration scores alone. However, when we integrated immune-related functions as additional features, we were able to accurately distinguish tumor tissues from normal ones in all 11 cancers. In terms of performance, the lowest accuracy corresponded to breast cancer (BRCA), which reaches 88%, and the highest accuracy hitting up to 99% corresponded to glioblastoma multiforme (GBM).

## Materials and methods

### Data collection

We obtained transcriptomic data of level 3 for 11 solid tumors from the TCGA database, as is shown in Table 1. The 11 cancers were selected according to differences in expression of HGF or c-MET genes between tumor and normal samples. The ComBat R package normalized the read count and eliminated batch processing effects [20]. Compared with a range of cancers, the specificity of each cancer type and data noise were avoided to some extent, facilitating subsequent analysis of the HGF/C- MET pathway risk genes that are stably present in cancer. We collected a list of genes relevant to immune regulation from the ImmPort database [21], involving 1811 genes. These genes were derived from molecules such as costimulatory molecules, chemokines and cytokines.

**Table 1:** Dataset information

Cancer	Tumor	Normal
LIHC	371	50
LUAD	515	59
BRCA	1097	114
ESCA	184	11
PRAD	497	52
HNSC	520	44
PAAD	178	4
GBM	158	5
THCA	505	59
CESC	303	3
COAD	286	41

The 11 cancer datasets used in this study. The first column indicates the type of cancer. The second and third columns correspond to the number of tumor or normal samples, respectively

### HGF/c-MET pathway activation sample identification

In samples affected by HGF/c-MET pathway activation, HGF was expected to be co-expressed with c-MET. Conversely, samples with unrelated HGF and c-MET expression were supposed to be more likely to belong to the group with an inactivated HGF/c-MET pathway. Expression of HGF and c-MET in all samples was scaled from 0 to 1 so that the ratio of the two genes in samples with activated HGF/c-MET pathway is close to 1. We took samples with a rate between 0.5 and 1.5 as the activated HGF/c-MET pathway group, and the others as the inactivated group.

### HGF/c-MET-related gene recognition

After obtaining the activated HGF/c-MET group and the inactivated HGF/c-MET group, immune-related genes that were significantly differentially expressed between the two groups were screened utilizing the Limma algorithm [22]. These genes were thought to be downstream genes differentially expressed after activation of the HGF/c-MET signaling pathway. Since we combined 11 cancers, some genes may be differentially expressed only in some samples considering the heterogeneity of cancer, and thus missed by differential analysis. Hence, we did not use the log_2_FC as a screening criterion. Instead, we selected genes with *p*-values < 0.05 as differentially expressed genes.

### Functional enrichment analysis

We used the statistical method of the clusterProfiler R package [23] to conduct a functional annotation analysis on HGF/c-MET-related immunoregulatory genes and identify their potential regulatory functions. Since the genes we selected were all immunoregulatory genes, the enriched biological functions were highly concentrated in the immune-related pathways, allowing us to identify and explain the molecular mechanisms of the HGF/c-MET pathway more precisely from the perspective of immune regulation.

### Functional pathway immune score

Differences in gene expression are apparent at different stages, and the genes that are functionally related to each other are concentrated in the same pathway. Therefore, based on the expression of the differentially expressed genes in each pathway, the overall deviation score for the pathway was calculated according to Eq. 1 [24].

1 $$ A\left( P \right) = log_{2} \left( {\frac{{\sqrt {\mathop \sum \nolimits_{i = 1}^{m} \omega_{i} \left( {X_{i} - \mu_{i} } \right)^{2} } }}{{\sqrt {\mathop \sum \nolimits_{j = 1}^{n} \omega_{j} \left( {X_{j} - \mu_{j} } \right)^{2} } }}} \right) $$

For the functional term *P*, *A (P)* is the function of the imbalance score, *m* is the number of differentially expressed genes needed for the pathway to increase, *n* is the number of differentially expressed genes required for the pathway to decrease, *ω* is the network weight in co-expression of the gene, *X*_*i*_ is the uptake of gene i’s expression value, *X*_*j*_ is the expression value of gene j, and *μ* is the mean value of the expression of the gene in the stage I sample; log_2_ transformation of the whole expression was taken. If *A (P)* = 0, the upregulated and downregulated gene achieves equilibrium. If *A (P)* is > 0, the upregulated gene is dominant and the function has an upward bias. If *A (P)* is < 0, the downregulated gene is dominant in the pathway and the function will have an occurrence of downward bias. Then we performed 1000 times of permutation procedure and in each cycle the same number of genes were randomly selected from the gene pool computing the random deviation score. The degree score (DS) of path *P* from the normal state is calculated using Eq. 2. ${\mu }^{^{\prime}} and {sd}^{^{\prime}}$ represent the mean and standard deviation of 1000 times permutation.

2 $$ DS\left( P \right) = \left( {\frac{{{\text{A}}\left( P \right) - \mu^{\prime}}}{{sd^{^{\prime}} }}} \right) $$

### Immune infiltration analysis

To unravel the downstream functions of the HGF/c-MET signaling pathway and explain the underlying mechanisms of cancers’ diverse prognosis, we used the expression of HGF/c-MET-related immune genes and the CIBERSORT algorithm [25] to assess immune cell infiltration. According to the immune score, the degree of activation of each immune-related pathway in any sample could be evaluated. The immune cell infiltration analysis facilitated the comparison of the differences in cellular components of different samples and immune cells in different pathways, thus analyzing how various immune cell components were changed after the HGF/c-MET pathway was activated.

### Tumor diagnostic model

Using the immunological scoring of immune-related pathways and immune cell infiltration ratio, we combined the machine learning algorithms for feature selection. We screened immune cells and pathways that are significantly associated with at least one cancer. We utilized the deep learning algorithm to build a neural network [26] and conducted cross-validation and accuracy assessment. All normal tissue samples from all the 11 cancers data were collected as a control group, and models were employed to predict tumor samples and control groups for each type of cancer. Finally, an ROC curve was used to evaluate the prediction accuracy of the model for different cancers.

### Survival analysis

The HGF/c-MET pathway is significantly associated with tumor cell development, and its downstream pathway can be used to distinguish tumors from normal samples accurately. We hope to further study the relationship between HGF/c-MET and cancer prognosis. Therefore, we used the survival R package [27] to evaluate the relationship between HGF, c-MET and other immune cell infiltration scores and survival prognosis for each cancer.

#### Cell culture

The human kidney cancer cell lines A549, H40, EC109, KYSE450 were purchased from the American Type Culture Collection (ATCC; Manassas, VA, USA) and cultured in DMEM supplemented with 10% fetal bovine serum, 100 U/mL penicillin, and 100 μg/mL streptomycin. All cells were maintained at 37°C in 5% CO_2_ atmosphere.

#### Silencing of IQGAP by small interfering RNA

The siRNA (purchase from Shanghai Gene Pharma) targeting position 5′- GGCCAUGAAUUUGACCUCUAUGAAA-3′, 5′- GGUGGGAUUCCUGCAUUCCUCUCAU-3′ of human HGF and c-MET mRNA were synthesized. A nonspecific scramble siRNA was used as negative control (NC). The final concentration of siRNAs is 100 nM. The siRNAs were transiently transfected into cells using Lipofectamine 3000 (Invitrogen) according to the manufacturer’s instruction. Assays were performed 48 h after transfection.

## Results

### Data collection

**Fig. 1:**
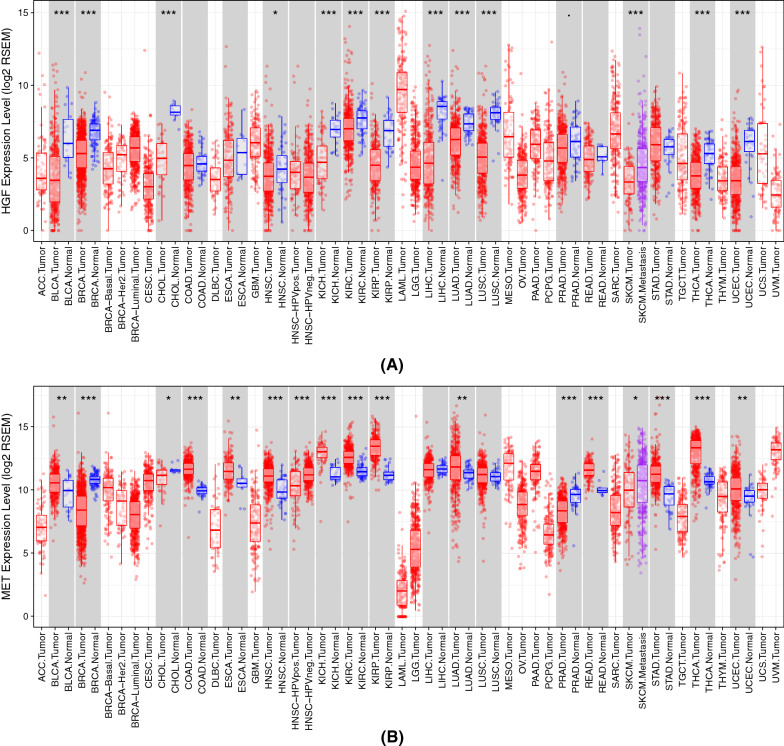
Distribution of HGF and c-MET genes in TCGA datasets. The red and blue bars indicate tumor and normal samples, respectively. Some tumors consist of multiple subtypes such as BRCA and HNSC and, thus, may have multiple bars. Stars on the top signify that the expression level between tumor and normal samples is diverse

We downloaded the RNAseq data for 11 solid tumors from the TCGA database, as is shown in Table 1. All data include tumor tissue samples and normal tissue samples as well as corresponding expression data for 20,530 genes. After removing the batch effect using the ComBat R package, we combined 11 datasets of cancer data, including 4182 tumor samples and 442 normal tissue samples from 11 cancers. We compared the expression profiles of HGF and c-MET in different cancer samples and corresponding normal samples in the TCGA database, as is shown in Fig. 1. It can be intuitively observed from the boxplot that HGF and c-MET are significantly differentially expressed in almost all cancer samples. We selected 11 significant solid tumors as the analytical data for this study, as is shown in Table 1.

### Identification of samples with activated HGF/c-MET pathway

**Fig. 2:**
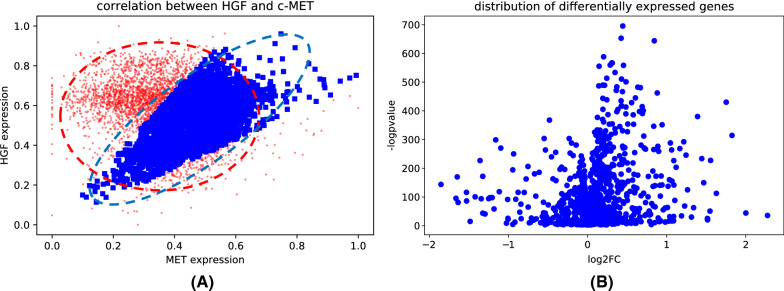
Activated samples and differentially expressed genes. **A** In activated samples HGF and c-MET are supposed to be correlated, as shown in blue. Samples marked in red are considered inactivated. **B** The X axis is log2FC and the Y axis is the log-transformed p-values. Even though most genes have a relatively small fold change, the p-values are extremely significant

In the HGF/c-Met pathway group, the expression interval of HGF/ c-Met was modified to 0–1, and the expression ratio of the two genes was close to 1. By screening samples with ratios > 0.5 and < 1.5, we identified 2852 activated samples and 2241 inactivated samples. Based on the Pearson correlation coefficient, the correlation coefficient between the two genes was 0.63 and the *p*-value was 2.32e−264 in the samples with activated HGF/c-MET pathway. The correlation profile is shown in Fig. 2A.

As is shown in Fig. 2A, the expression profiles of the HGF and MET genes in all samples show distinctly different patterns. Red dots represent inactivated samples and blue dots represent the activated samples. In the activated samples, as the expression level of the gene HGF increases, the expression level of the gene c-MET increases correspondingly.

### HGF/c-MET-related gene recognition

We used the correlation between HGF and c-MET to split the sample into activated and inactivated groups. Combined with the differential analysis, the genes with *p*-values < 0.05 were selected as the HGF/c-MET signaling related genes. In the end, we screened out 755 upregulated genes and 395 downregulated genes. We also visualized the distribution of log_2_FC and negative logarithmically transformed *p*-values of differentially expressed genes, as is shown in Fig. 2B.

In Fig. 2B, the horizontal axis is log_2_FC, and the vertical axis is the negative logarithmically transformed p-value, and each dot represents a differentially expressed gene. As the distribution indicates, the fold change of some differentially expressed genes is close to 0, but the corresponding *p*-values are very significant. It demonstrates that although some genes have no significant difference in terms of the overall mean or median, they are significantly differentially expressed in the subgroup of patients, thus obtaining significant *p*-values.

### Functional enrichment analysis

**Fig. 3:**
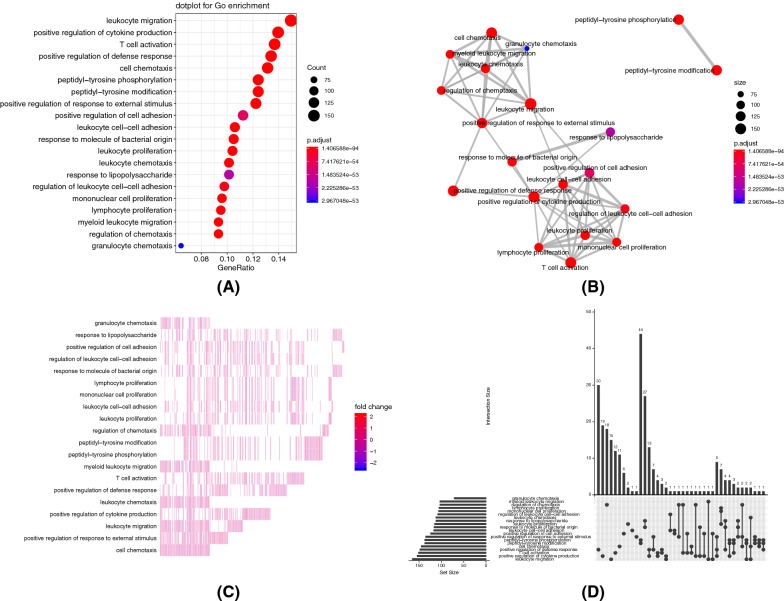
Functional enrichment. **A** Dot plot of the top 20 functions. The color indicates the significance and the size indicates the counts of involved genes. **B** Emap plot of functions in which the nodes represent functions and the connections represent shared genes. **C** Heatmap of differentially expressed genes in enriched functions. **D** Upset plot of functions exhibiting the shared genes among functions

We screened immune-related genes that were significantly differentially expressed between activated and inactivated HGF/c-MET pathway. To further clarify the functions regulated by these differential genes, we conducted a functional enrichment analysis, which is shown in Fig. 3.

The results of functional enrichment analysis revealed that downstream genes related to the activation of the HGF/c-MET pathway were mainly involved in the regulation of immune cell proliferation, migration and intercellular interactions (Fig. 3A). It is worth noting that these functions were highly linked to each other, suggesting that differentially expressed immune-related genes were involved in the regulation of multiple similar or related biological functions (Fig. 3B). Among these enriched functions, peptidyl−tyrosine phosphorylation, peptidyl−tyrosine modification and response to lipopolysaccharide shared most genes (Fig. 3D) with leukocyte proliferation. At the same time, the differentially expressed genes showed a significant mutual exclusion pattern, that is, specific genes specifically regulated a particular function (Fig. 3C).

### Functional pathway immune score

**Fig. 4:**
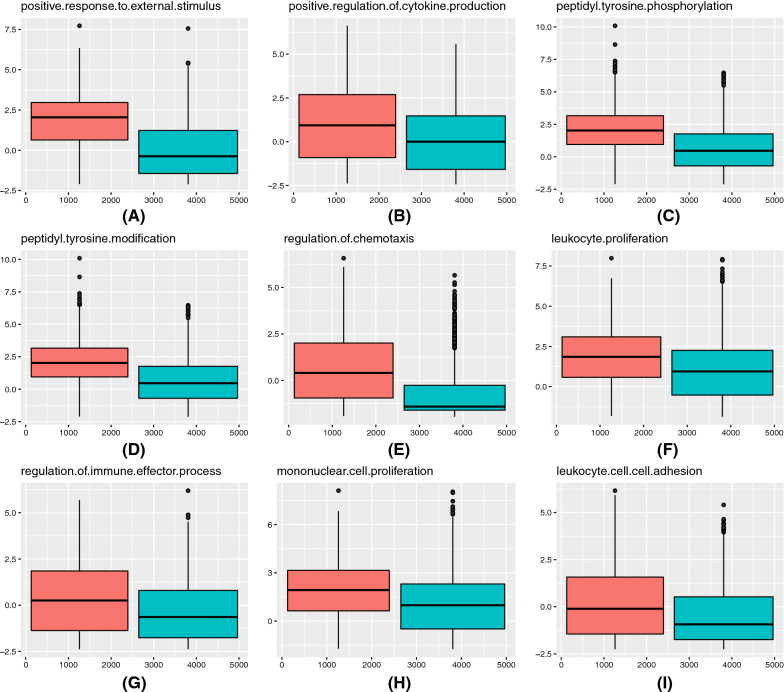
Pathway score distribution. The activated and inactivated samples are marked in red and blue, respectively. The X axis indicates the index of samples and the Y axis indicates the corresponding pathway score

We used the expression of immune-related genes in each enriched function to assess the immune scores of each function across all samples. By comparing the immune scores of each pathway between the activated and inactivated samples, we extracted the nine most significant pathways, as is shown in Fig. 4**.**

The following analysis by the student’s t-test, the *p*-values of the nine pathways, shown in Fig. 4, were all below 0.05, and all pathways were supposed to be upregulated in samples whose HGF/c-MET pathway was activated. These nine pathways were mainly involved in the positive response of the immune system.

### Immune cell infiltration analysis

**Fig. 5:**
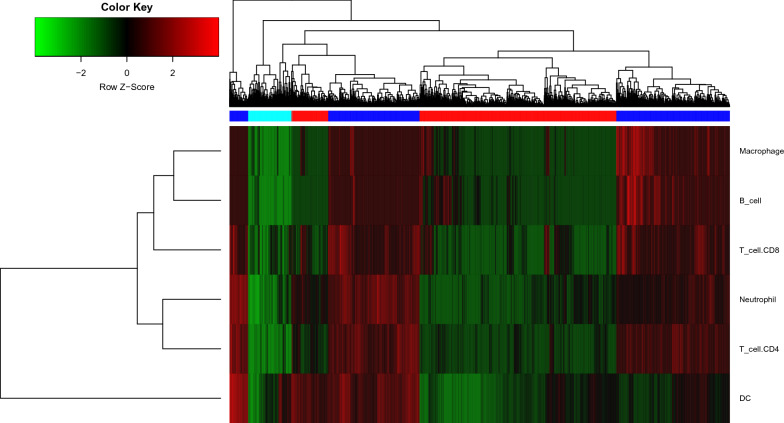
Cluster of immune infiltration. Each row represents one immune cell and each column represents one sample. The normal control, activated, and inactivated samples are marked in light blue, dark blue, and red, respectively

Through functional enrichment analysis and quantitative analysis of immune scores, we found that the immune response was significantly positively regulated when the HGF/c-MET pathway was activated. To clarify the proportion of different cellular components during the immune response, we used the CIBERSORT algorithm to quantify different immune cells. We calculated the infiltration fraction of six immune cells, including B cells, T cells, CD4^+^ T cells, CD8^+^ T cells, neutrophils, macrophages, and DCs in each sample. Using hierarchical clustering, we clustered the samples, as is shown in Fig. 5.

We found a significant difference between the activated and inactivated samples based on the immune cell infiltration score, with the immune cell infiltration fraction significantly increased in the activated group. In the inactivated group, the immune cell infiltration fraction was relatively low. However, it is difficult to distinguish between normal samples and tumor samples only relying on the immune cell infiltration fraction.

**Fig. 6:**
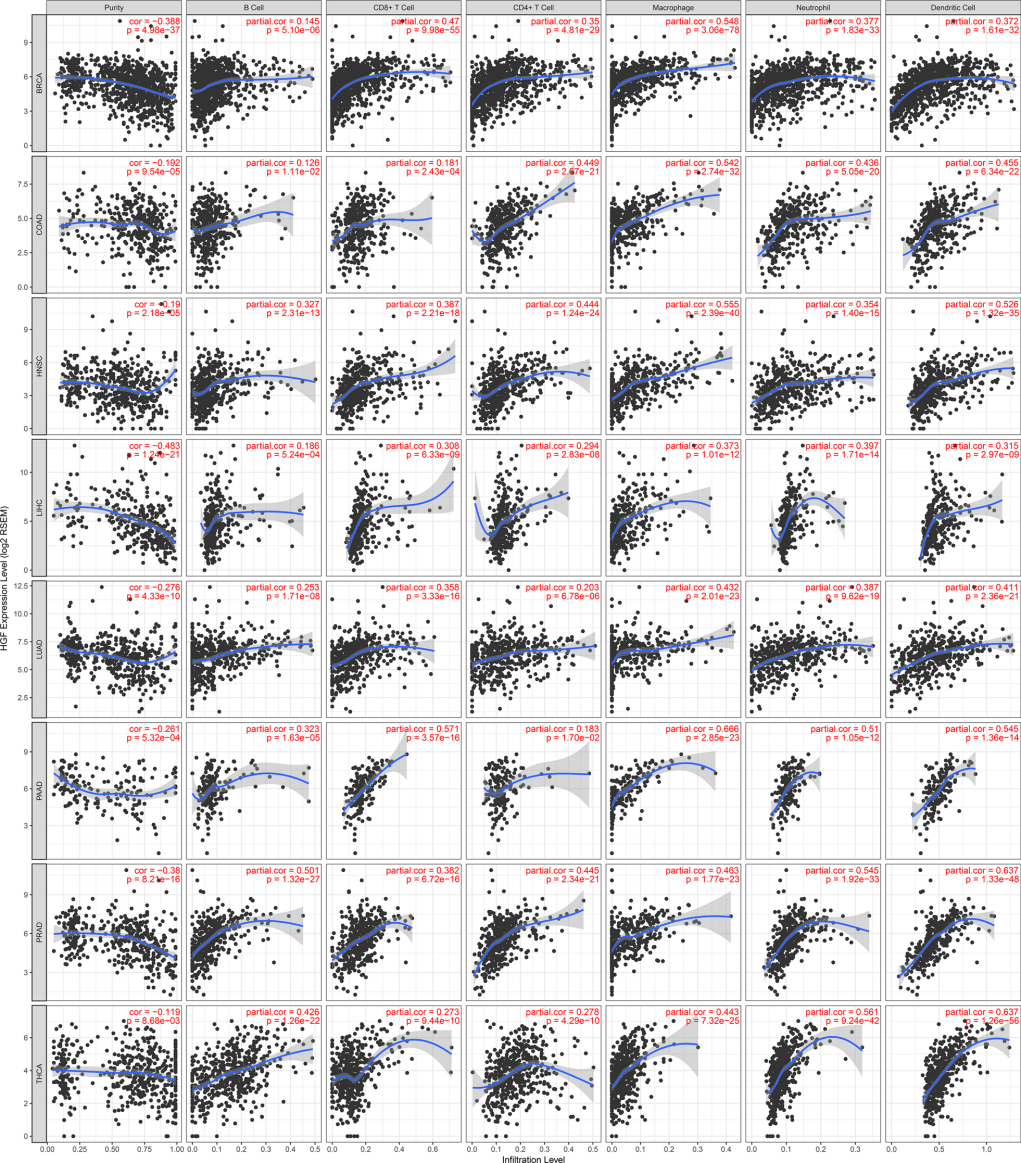
Correlation between HGF and six immune cells in 11 tumors. Each row is one type of tumor and the first column represents the purity of the tumor. The second to seventh columns represent correlations between each immune cell and HGF

**Fig. 7:**
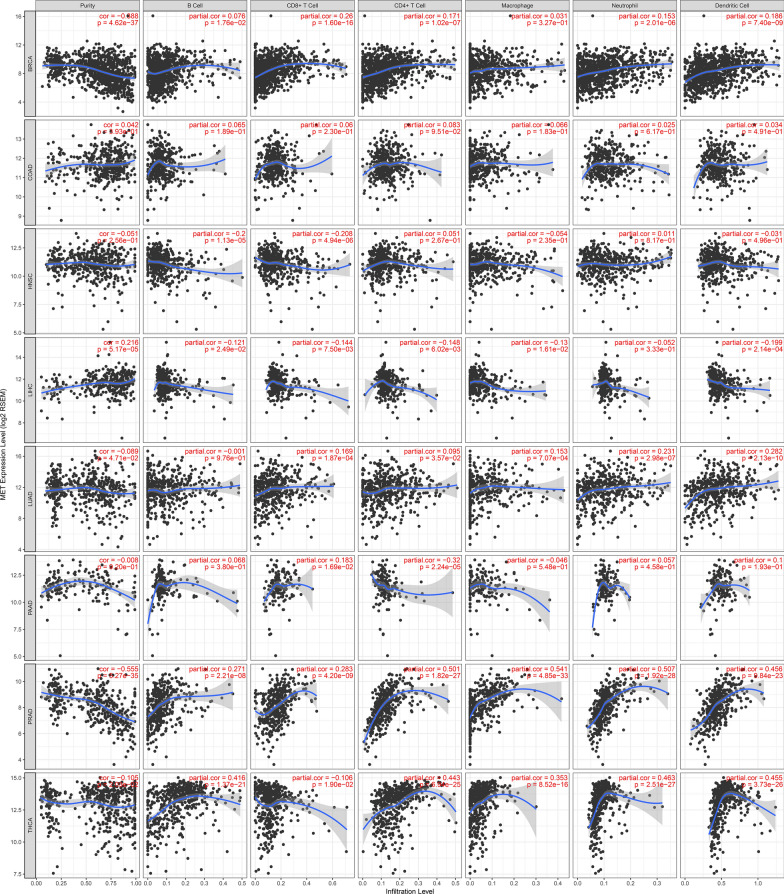
Correlation between MET and six immune cells in 11 tumors. Each row is one type of tumor and the first column represents the purity of the tumor. he second to seventh columns represent correlations between each immune cell and MET.

On the other hand, since we combined the data of 11 tumors, the infiltration of different immune cells in various tumors was also highly heterogeneous. To further clarify the correlation between the infiltration of each immune cell and the HGF or c-MET gene, a correlation analysis was performed, as is shown in Figs. 6**, **7, Additional file 1: Figure S1 and Additional file 2: Figure S2.

We found that different immune cells were differentially activated by HGF and c-MET in the 11 tumors. For example, in BRCA, adenocarcinoma of colon (COAD) and most other tumors, all the six immune cells showed a positive correlation with HGF, indicating that immune cells were activated or recruited by the HGF/c-MET pathway. However, no significant correlation was observed in GBM or cervical squamous cell carcinoma (CESC). Meanwhile, in the c-MET correlation analysis, we found that some immune cells showed a negative correlation with c-MET. This series of results demonstrated that the HGF/c-MET pathway played an essential role in the development of multiple tumors and activated downstream immune cells as well as immune-related pathways. However, for some tumors, such as GBM and CESC, there may be other mechanisms that are more dominant than the HGF/c-MET pathway.

### Tumor diagnostic model

**Fig. 8:**
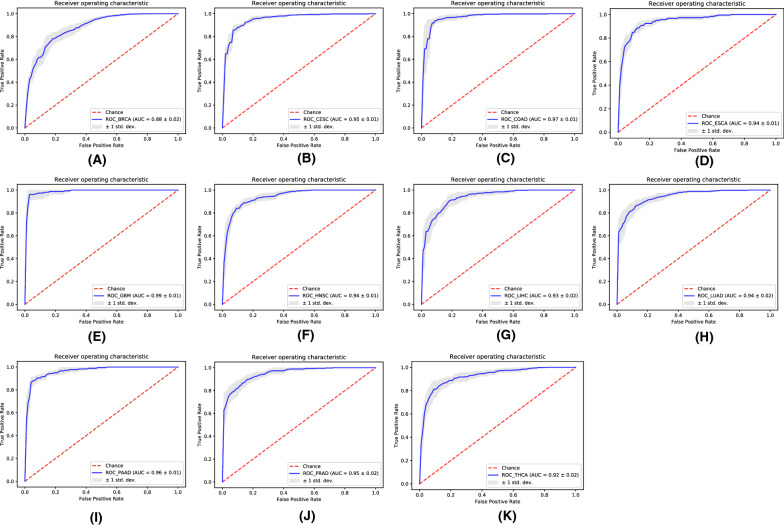
ROC performance of model for each type of cancer. Each ROC graph corresponds to one type of cancer. The red curve is the random curve. The X and Y axes are the false-positive rate and true-positive rate, respectively

Activation of the HGF/c-MET pathway plays a vital role in tumorigenesis. By intervening in the downstream immune cell pathway, it affects the TME and leads to tumorigenesis. Therefore, we hope to integrate the HGF/c-MET pathway and level of immune regulation to achieve tumor diagnosis and prediction. We collected six immune cells and the 20 significantly enriched immune pathways as features. Using the neural networks, we predicted each cancer separately, and the results are shown in Fig. 8.

Using the integrated immune cell infiltration fraction and the enrichment pathway immune scores can accurately distinguish tumor samples from normal tissue samples. The highest precision was observed in GBM, with an accuracy of 0.99, while the worst emerged in BRCA, with a precision of 0.88.

### Survival analysis

**Fig. 9:**
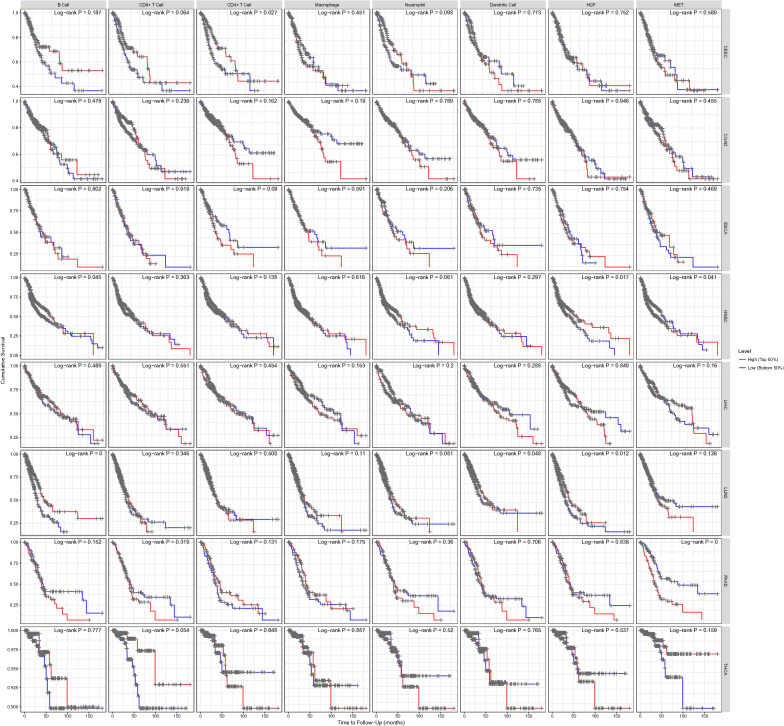
Survival analysis. Each row represents one type of cancer. Each column represents one variable, including HGF, MET, and the 6 immune cells

We used the survival R package for log-rank analysis and calculation of p-values. The results are shown in Fig. 9 and Additional file 3: Figure S3. The survival analysis shows that the prognosis of some tumors was significantly correlated with HGF/c-MET expression, including HNSC, lung adenocarcinoma (LUAD) and pancreatic adenocarcinoma (PAAD). Some cancers were also associated with the degree of immune cell infiltration, such as CESC, COAD and thyroid cancer (THCA). This result suggests that although the activation of the HGF/c-MET pathway upregulated the downstream immune signaling pathway, which recruited more immune cells, the degree of HGF/c-MET pathway or immune infiltration varies among different cancers in terms of prognosis. This is mainly caused by the various mechanisms, recurrence or metastasis of cancer. Therefore, to achieve a successful tumor diagnosis or prognosis assessment more comprehensively, systematic integration of the HGF/c-MET pathway and immune-related pathways are needed for further analysis.

#### HGF / C-MET silenced could suppress tumor proliferation and invasion

**Fig. 10:**
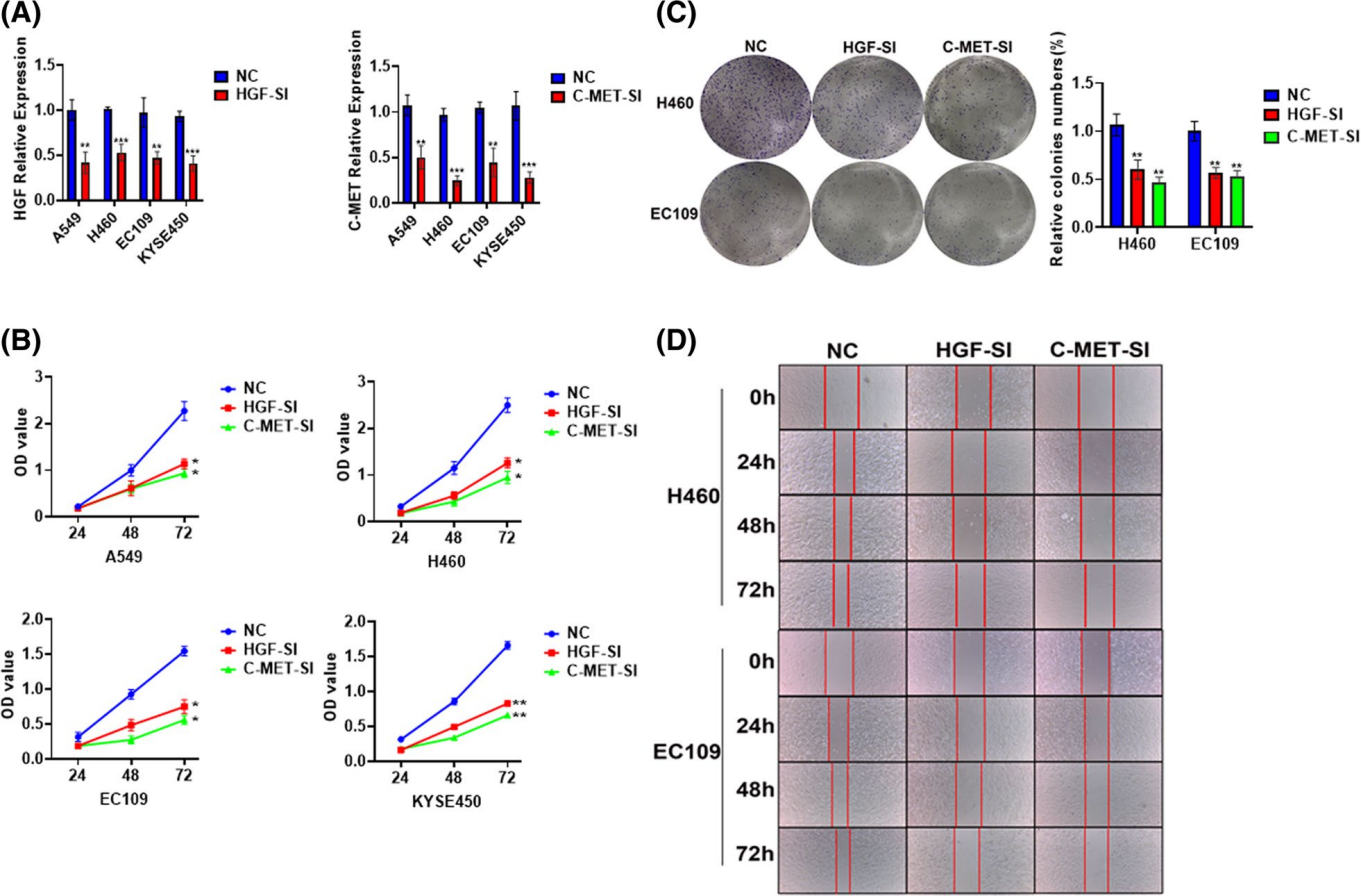
The expression of HGF and c-MET can affect the proliferation and invasion of lung cancer and esophageal cancer. **A** qRT-PCR analysis of HGF and c-MET expression after silencing the gene. **B** 500 cells were seeded in 6-well plates, and after 2 weeks of culture, representative images of foci formation in monolayer culture between NC, HGF-SI and c-MET-SI cells, and the number of colonies detected. **C** The cell proliferation rate between NC, HGFSI and c-MET-SI cells were measured by CCK8 assay. **D** Scratch test detects cell invasion ability between NC, HGF-SI and c-MET-SI cells. * represents *p* < 0.05, ** represents *p* < 0.01

In order to further explore the impact of the HGF/c-MET pathway on tumors, we conducted some experiments *in vitro* in lung cancer and esophageal cancer cell lines. We silenced HGF and c-MET genes respectively as shown in Fig. 10A, and we have done a CCK8 proliferation assay, colony and Wound-Healing assay. We found that whether HGF or c-MET was silenced, the proliferation and invasion of tumor cells would be inhibited as is shown in Fig. 10B–D. Our results demonstrated that the HGF/c-MET pathway could affect tumor proliferation and invasion.

## Discussion

With the development of bioinformatics, increasing attention has been focused on finding recurrent mechanisms in various cancers. A recurrent mechanism might be a driver gene, a core pathway or even a complex regulatory network. In this study, we integrated data from 11 different solid tumors, intending to find molecular mechanisms commonly applicable to tumors. Abnormal activation of the HGF/c-MET pathway and immune cell infiltration have been widely demonstrated to play an essential role in a variety of tumors, so we integrated HGF/c-MET pathway and immunoregulatory elements to analyze the underlying driving mechanisms of cancer. MET is a tyrosine kinase receptor involved in embryonic development, organogenesis, and wound healing. Hepatocyte growth factor/scatter factor (HGF/SF) and its alternative splicing isoforms (NK1 and NK2) are the only known ligands of the MET receptor. MET has high-level tissue specificity and is mainly expressed in epithelial-derived cells, whereas HGF is primarily expressed in mesenchymal-derived cells. When HGF binds to its cognate receptor MET, it induces MET dimerization. The specific biological mechanism behind this process is still unclear. Abnormal MET activation in cancer is associated with poor prognosis. Possible reasons include that MET activation triggers tumor growth, angiogenesis or metastasis. Generally, only stem and progenitor cells express MET, which enables these cells to grow invasively. The activation of MET also helps produce new tissues in the embryo or regenerate damaged tissue in adulthood.

HGF/c-Met signaling dysfunction has been reported to be related to cell proliferation, progression and metastatic characteristics of several tumor types, including COAP, which suggests that it has potential value as a novel therapeutic target. Although c-MET activation is transient during physiological events, c-MET signaling may be constitutively active during tumor onset and progression. Activating c-MET pathways in tumor cells during tumor progression enhances the ability to disaggregate from surrounding tumor cells, which further destroys the basement membrane and improves cell mobility and metastatic risk.

It has been suggested that changes in tumor cells can benefit from the changes of TME in terms of enhancing proliferation and increasing chemoresistance. The immune cell infiltration is one of the main factors to interfere with the TME.

The immune system has been validated to play a dual role in the internal environment, which is known as the executor of cancer immunoediting [28]. Generally, the immune system eliminates cancer cells or inhibits the growth of cancer cells, but in certain conditions, the immune system promotes tumor progression by interfering with the TME or recruiting more resistant cancer cells. Cellular components in the TME include fibroblasts, adipocytes, neural and neuroendocrine cells, endothelial cells, pericytes and mesenchymal stem cells, the most prominent of which are lymphocytes and myeloid populations, including T cells, B cells, NK cells, macrophages, and DCs. The immune cells infiltration varies across different cancers. For example, in GBM, the degree of infiltration of all immune cells is significantly higher than in esophageal carcinoma (ESCA). The degree of infiltration of CD4^+^ T cells in THCA is considerably higher than that of other cell types. This also indicates that, to some extent, there are significant differences in the levels of immune regulation among different cancers. To quantify this difference in functional levels, we used a functional pathway immune scoring algorithm to score the enriched immune pathways.

In the present study, we divided the samples into an activated group and an inactivated group based on the expression correlation of HGF/c-MET and then extracted immune-related genes differentially expressed between the groups. Through functional enrichment analysis, we found that these genes were mainly involved in mediating immune cell proliferation, migration and intercellular interaction. Through the pathway immune scoring algorithm, we quantified the enriched functional pathways. Using immune gene expression profiles, we evaluated the infiltration fraction of 6 immune cells. Finally, we integrated HGF/c-MET, immune cell infiltration fraction, and immune pathway score as features and predicted 11 tumors by constructing a neural network model. Among the 11 tumors, the model had the best predictive performance on GBM with an accuracy of 99%. The prediction of BRCA was the worst, still reached 88%. The probable cause is that BRCA contains multiple different subtypes. There are significant differences in the levels of immune regulation between the different subtypes; hence, the model fails to achieve optimal performance when predicting the overall BRCA. However, if BRCA patients are to be diagnosed based on subtypes, a better accuracy should be obtained.

In addition to distinguishing between tumors and normal samples, we also attempted to compare the relationship between HGF/c-MET, immune infiltration and survival outcomes in patients with cancer. We found a significant correlation between immune infiltration and survival prognosis in CESC, ESCA, LUAD, and PAAD. In long-lived patients in COAD and THCA, immune infiltration and survival prognosis were significantly associated. Besides, in HNSC, LUAD and PAAD, the expression of HGF/c-MET also determines the survival prognosis. These results further confirm that although HGF/c-MET abnormal activation and immune regulation abnormalities play an important role in the development of cancer, their effects vary in different cancers. This indicates the specificity of immunoregulatory abnormalities during the progression of different types of cancer. Therefore, it is critical to achieve a cancer-specific treatment and diagnosis for various tumors.

In this study, we systematically analyzed 11 different cancers, including expression correlation, immune infiltration, tumor diagnosis and survival prognosis from HGF/c-MET pathway and immune regulation, two biological mechanisms that have received extensive attention in cancer analysis. In contrast, we have found that it can be widely used in a variety of cancers to achieve tumor diagnosis. We found that HGF/c-MET and immune regulation levels are highly specific in different cancers. Therefore, although the HGF/c-MET pathway, immune cell infiltration and immune pathway scores integrated in this study can satisfy the prediction of 11 cancers, it is difficult to find a feature that could be widely used in all cancers. In contrast, a large number of studies have shown that HGF/c-MET activation affects cancer prognosis, but we found a significant relationship only in a small number of cancers by comparing HGF/c-MET expression and survival prognosis in 11 cancer patients.

Nevertheless, a correlation can be observed in some long-lived patients. Frankly, our study still has some limitations, including the fact that the entire research focuses on transcriptomic data. Other omics data for genes can play a more dominant role in certain cancers, including mutation, copy number variant, gene fusion and methylation. In subsequent studies, integrating the abovementioned omics data to augment the feature set may lead to a more specific and sensitive diagnostic model.

## Conclusion

We found that the HGF/c-MET pathway affected the TME mainly by interfering with the expression levels of other genes. Immune infiltration was another crucial factor involved in changes to the TME. The downstream immune-related genes activated by the HGF/c-MET pathway regulated immune-related pathways, which in turn affected the degree of infiltration of immune cells. Immune infiltration was significantly associated with cancer development and prognosis.

## Supplementary Information

**Additional file 1:** Correlation between HGF and six immune cells in GBM, ESCA, CESC.

**Additional file 2:** Correlation between c-MET and six immune cells in GBM, ESCA, CESC.

**Additional file 3:** The influence of infiltration of each immune cell and the expression on survival in BRCA, GBM, PRAD.

## Data Availability

All datasets generated for this study are included in the manuscript/Additional file 1, 2 and 3.

## Abbreviations

HGF: Hepatocyte growth factor
C-MET: c-mesenchymal-epithelial transition
TME: Tumor microenvironment
NSCLC: Non-small cell lung cancer
HCC: Hepatocellular carcinoma
CTLs: Cytotoxic T-lymphocytes
TAMs: Tumor-associated macrophages
TFH: T follicular helper cells

## References

[1] Konstorum A, Lowengrub JS (2018). Activation of the HGF/c-Met axis in the tumor microenvironment: a multispecies model. J Theor Biol.

[2] Boromand N, Hasanzadeh M, ShahidSales S, Farazestanian M, Gharib M, Fiuji H, Behboodi N, Ghobadi N, Hassanian SM, Ferns GA (2018). Clinical and prognostic value of the C-Met/HGF signaling pathway in cervical cancer. J Cell Physiol.

[3] Granito A, Guidetti E, Gramantieri L (2015). c-MET receptor tyrosine kinase as a molecular target in advanced hepatocellular carcinoma. J Hepatocell Carcinoma.

[4] Arnold L, Enders J, Thomas SM (2017). Activated HGF-c-Met axis in head and neck cancer. Cancers.

[5] Stanley A, Ashrafi GH, Seddon AM, Modjtahedi H (2017). Synergistic effects of various Her inhibitors in combination with IGF-1R, C-MET and Src targeting agents in breast cancer cell lines. Sci Rep.

[6] Lam BQ, Dai L, Qin Z (2016). The role of HGF/c-MET signaling pathway in lymphoma. J Hematol Oncol.

[7] Krause DS, Van Etten RA (2005). Tyrosine kinases as targets for cancer therapy. N Engl J Med.

[8] Mo HN, Liu P (2017). Targeting MET in cancer therapy. Chronic Dis Transl Med.

[9] Hu CT, Wu JR, Cheng CC, Wu WS (2017). The therapeutic targeting of HGF/c-Met signaling in hepatocellular carcinoma: alternative approaches. Cancers.

[10] Bradley CA, Salto-Tellez M, Laurent-Puig P, Bardelli A, Rolfo C, Tabernero J, Khawaja HA, Lawler M, Johnston PG, Van Schaeybroeck S (2017). Targeting c-MET in gastrointestinal tumours: rationale, opportunities and challenges. Nat Rev Clin Oncol.

[11] Xu X, Zhu Y, Liang Z, Li S, Xu X, Wang X, Wu J, Hu Z, Meng S, Liu B (2016). c-Met and CREB1 are involved in miR-433-mediated inhibition of the epithelial-mesenchymal transition in bladder cancer by regulating Akt/GSK-3beta/Snail signaling. Cell Death Dis.

[12] Furge KA, Zhang YW, Vande Woude GF (2000). Met receptor tyrosine kinase: enhanced signaling through adapter proteins. Oncogene.

[13] Wang W, Dong J, Wang M, Yao S, Tian X, Cui X, Fu S, Zhang S (2018). miR-148a-3p suppresses epithelial ovarian cancer progression primarily by targeting c-Met. Oncol Lett.

[14] Demkova L, Kucerova L (2018). Role of the HGF/c-MET tyrosine kinase inhibitors in metastasic melanoma. Mol cancer.

[15] Pasquini G, Giaccone G (2018). C-MET inhibitors for advanced non-small cell lung cancer. Expert Opin Investig Drugs.

[16] Christensen JG, Burrows J, Salgia R (2005). c-Met as a target for human cancer and characterization of inhibitors for therapeutic intervention. Cancer Lett.

[17] Goc J, Fridman WH, Sautes-Fridman C, Dieu-Nosjean MC (2013). Characteristics of tertiary lymphoid structures in primary cancers. Oncoimmunology.

[18] Pages F, Berger A, Camus M, Sanchez-Cabo F, Costes A, Molidor R, Mlecnik B, Kirilovsky A, Nilsson M, Damotte D (2005). Effector memory T cells, early metastasis, and survival in colorectal cancer. N Engl J Med.

[19] Naito Y, Saito K, Shiiba K, Ohuchi A, Saigenji K, Nagura H, Ohtani H (1998). CD8+ T cells infiltrated within cancer cell nests as a prognostic factor in human colorectal cancer. Cancer Res.

[20] Somekh J, Shen-Orr SS, Kohane IS (2019). Batch correction evaluation framework using a-priori gene-gene associations: applied to the GTEx dataset. BMC bioinformatics.

[21] Bhattacharya S, Andorf S, Gomes L, Dunn P, Schaefer H, Pontius J, Berger P, Desborough V, Smith T, Campbell J (2014). ImmPort: disseminating data to the public for the future of immunology. Immunol Res.

[22] Costa-Silva J, Domingues D, Lopes FM (2017). RNA-Seq differential expression analysis: An extended review and a software tool. PloS One.

[23] Yu G, Wang LG, Han Y, He QY (2012). clusterProfiler: an R package for comparing biological themes among gene clusters. Omics.

[24] Wu T, Wang Y, Jiang R, Lu X, Tian J (2017). A pathways-based prediction model for classifying breast cancer subtypes. Oncotarget.

[25] Chen B, Khodadoust MS, Liu CL, Newman AM, Alizadeh AA (2018). Profiling Tumor Infiltrating Immune Cells with CIBERSORT. Methods Mol Biol.

[26] Scalabrini Sampaio G, Vallim Filho ARA, da Santos Silva L, da Augusto Silva L (2019). Prediction of motor failure time using an artificial neural network. Sensors.

[27] Williams C, Lewsey JD, Briggs AH, Mackay DF (2017). Cost-effectiveness Analysis in R using a multi-state modeling survival analysis framework: a tutorial. Med Decis Making.

[28] Schreiber RD, Old LJ, Smyth MJ (2011). Cancer immunoediting: integrating immunity's roles in cancer suppression and promotion. Science.

