# Correction to: Molecular mechanism study of HGF/c-MET pathway activation and immune regulation for a tumor diagnosis model

**DOI:** 10.1186/s12935-021-02359-z

**Published:** 2021-12-14

**Authors:** Zhibo Shen, Wenhua Xue, Yuanyuan Zheng, Qishun Geng, Le Wang, Zhirui Fan, Wenbin Wang, Ying Yue, Yunkai Zhai, Lifeng Li, Jie Zhao

**Affiliations:** 1 Department of Pharmacy, The First Affiliated Hospital of Zhengzhou University, Zhengzhou, 450052 Henan People’s Republic of China; 2 Cancer Center, The First Affiliated Hospital of Zhengzhou University, Zhengzhou, 450052 Henan People’s Republic of China; 3 Department of Otorhinolaryngology, The First Affiliated Hospital of Zhengzhou University, Zhengzhou, 450052 Henan People’s Republic of China; 4 Department of Traditional Chinese Medicine, The First Affiliated Hospital of Zhengzhou University, Zhengzhou, 450052 Henan People’s Republic of China; 5 Internet Medical and System Applications of National Engineering Laboratory, Zhengzhou, China; 6 Department of Clinical Laboratory, The No.7. People’s Hospital of Zhengzhou, Zhengzhou, 450016 Henan China

## Correction to: Cancer Cell Int (2021) 21:374 10.1186/s12935-021-02051-2

In this article [1] the wrong figure appeared as Fig. 10c; the correct Fig. 10 should have appeared as shown in this erratum.

**Fig. 10:**
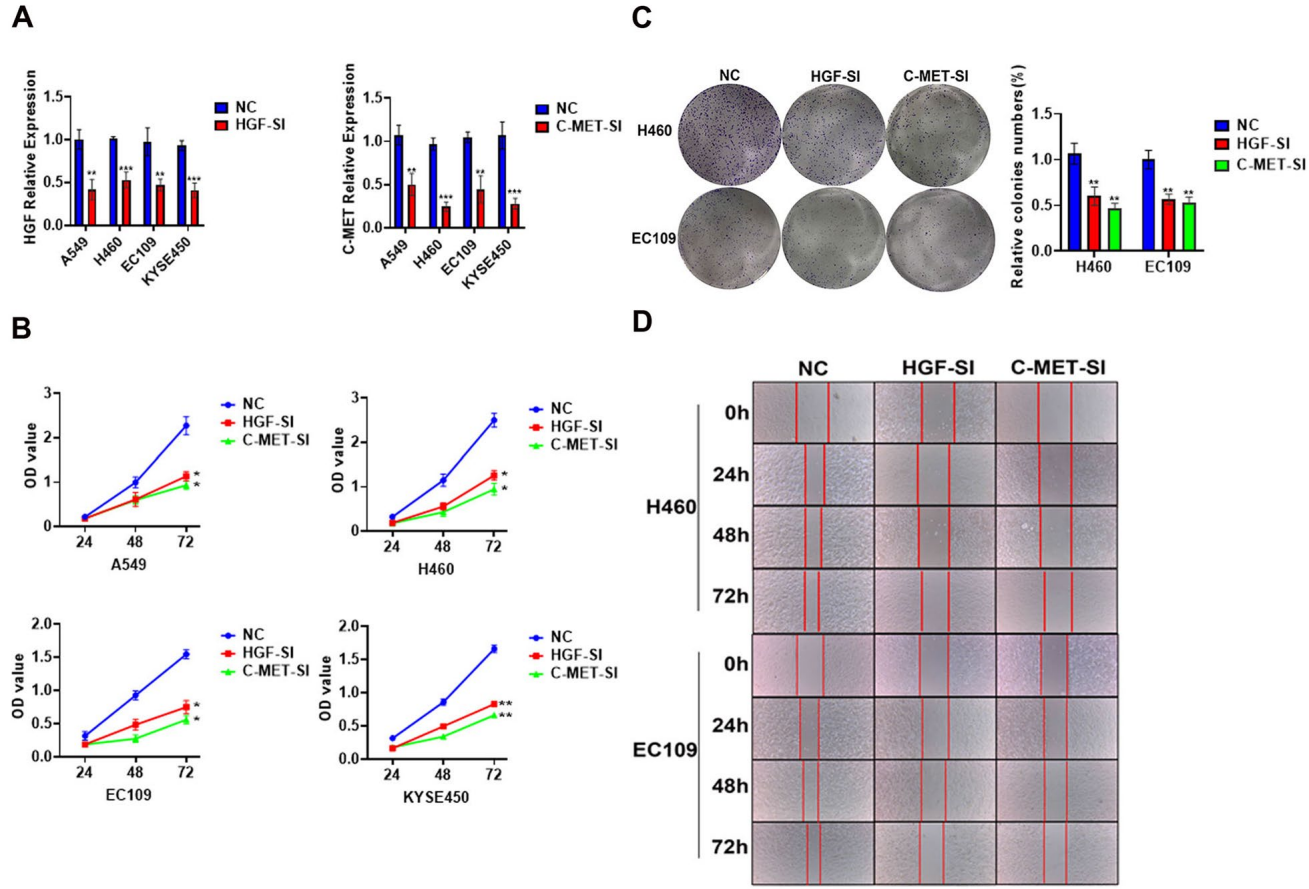
The expression of HGF and c-MET can affect the proliferation and invasion of lung cancer and esophageal cancer. **A** qRT-PCR analysis of HGF and c-MET expression after silencing the gene. **B** 500 cells were seeded in 6-well plates, and after 2 weeks of culture, representative images of foci formation in monolayer culture between NC, HGF-SI and c-MET-SI cells, and the number of colonies detected. **C** The cell proliferation rate between NC, HGFSI and c-MET-SI cells were measured by CCK8 assay. **D** Scratch test detects cell invasion ability between NC, HGF-SI and c-MET-SI cells. * Represents *p* < 0.05, ** represents *p* < 0.01
