# Supplementary figures and images for: Molecular mechanism study of HGF/c-MET pathway activation and immune regulation for a tumor diagnosis model
Source: Cancer Cell Int. 2021 Jul 14;21:374. doi: 10.1186/s12935-021-02051-2 (PMC8278741; doi:10.1186/s12935-021-02051-2)

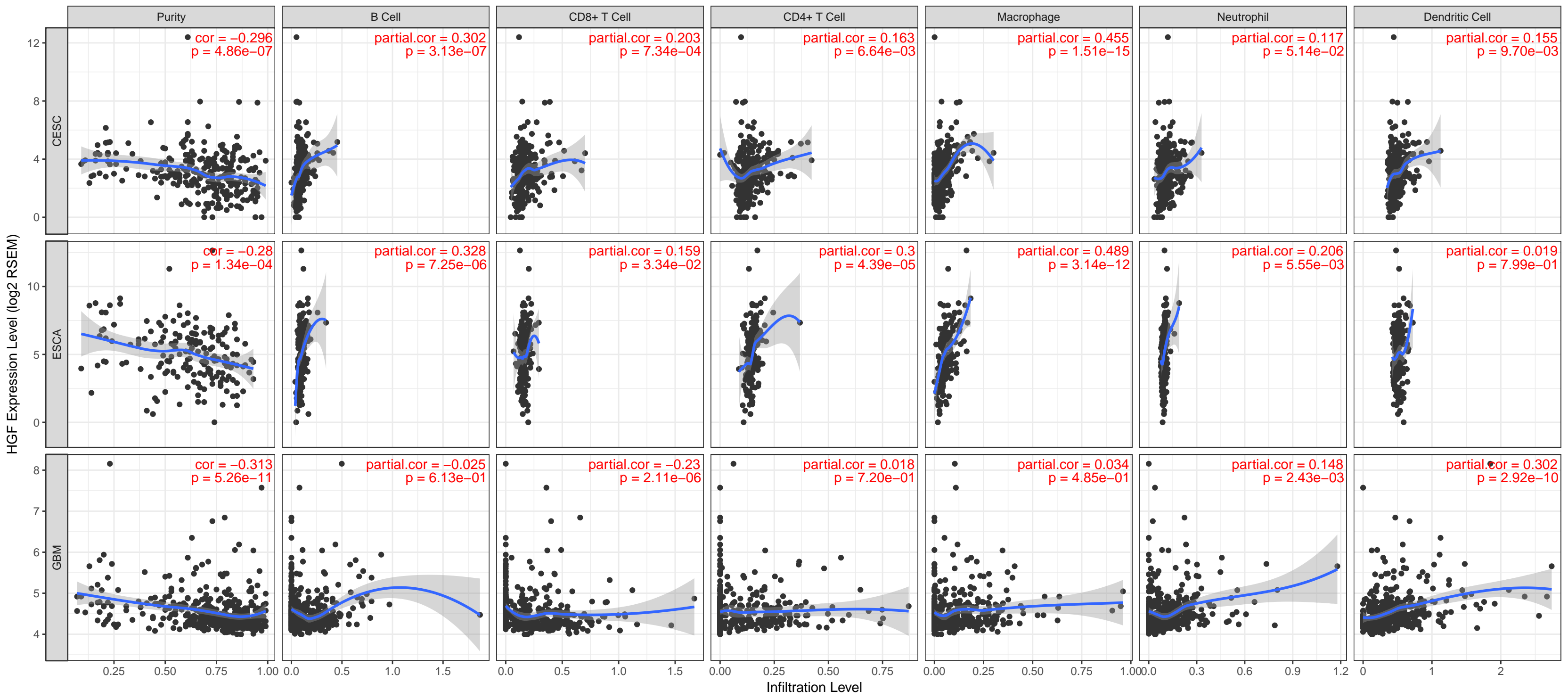

Supplement: Supplementary file 1 — Additional file 1: Correlation between HGF and six immune cells in GBM, ESCA, CESC. [file 12935_2021_2051_MOESM1_ESM.pdf]

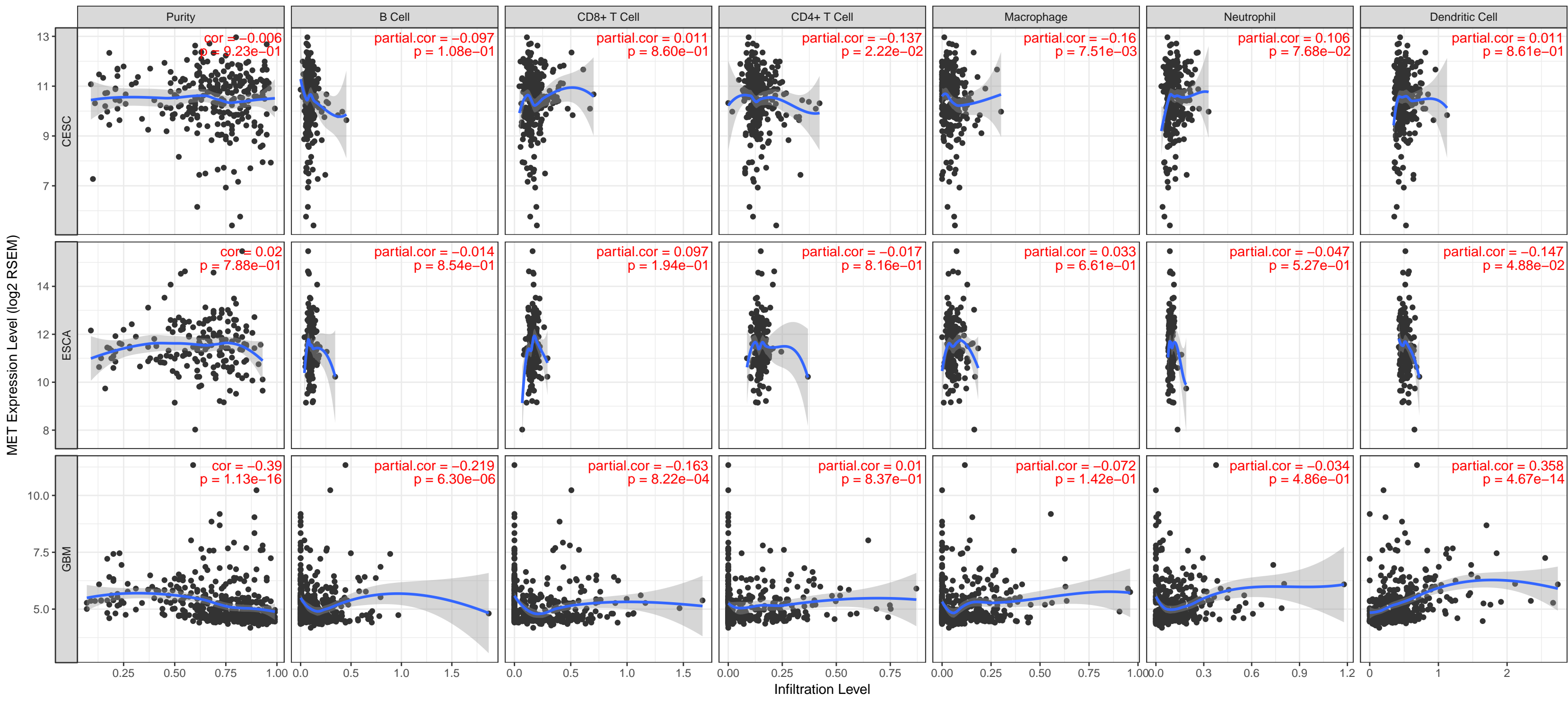

Supplement: Supplementary file 2 — Additional file 2: Correlation between c-MET and six immune cells in GBM, ESCA, CESC. [file 12935_2021_2051_MOESM2_ESM.pdf]

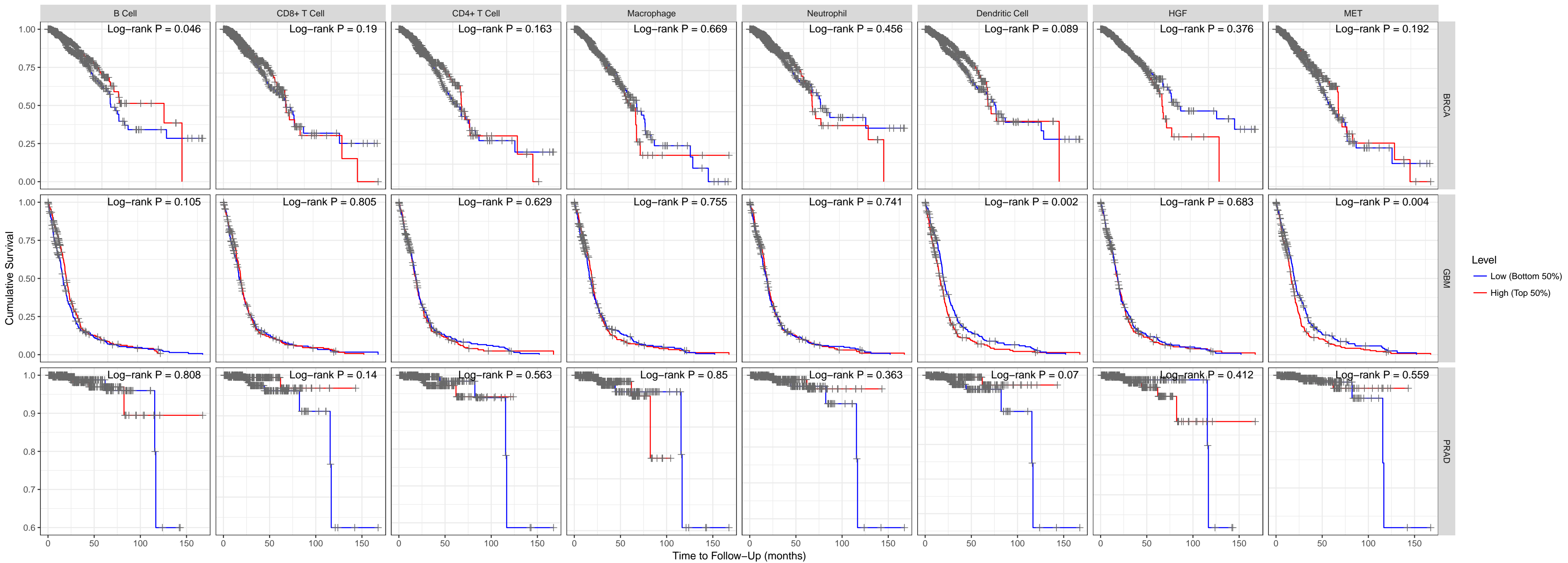

Supplement: Supplementary file 3 — Additional file 3: The influence of infiltration of each immune cell and the expression on survival in BRCA, GBM, PRAD. [file 12935_2021_2051_MOESM3_ESM.pdf]
